# ROCK1 activation-mediated mitochondrial translocation of Drp1 and cofilin are required for arnidiol-induced mitochondrial fission and apoptosis

**DOI:** 10.1186/s13046-020-01545-7

**Published:** 2020-02-19

**Authors:** Jinjiao Hu, Hongwei Zhang, Jie Li, Xiuxing Jiang, Yanhao Zhang, Qin Wu, liwen Shen, Jingshan Shi, Ning Gao

**Affiliations:** 1 College of Pharmacy, Army Medical University, 30 Gaotanyan Street, Shapingba District, Chongqing, 400038 China; 2 Key Laboratory of Basic Pharmacology of Ministry of Education and Joint International Research Laboratory of Ethnomedicine of Ministry of Education, Zunyi Medical University, Zunyi, China

**Keywords:** ROCK1, Cofilin, Drp1, Arnidiol, Mitochondrial fission, Apoptosis

## Abstract

**Background:**

Arnidiol is a pentacyclic triterpene diol that has multiple pharmacological activities. However, the apoptotic activities of arnidiol in human cancer cells have not yet been explored, nor has the mechanism by which arnidiol induces apoptosis been examined in depth.

**Methods:**

MDA-MB-231 cells and xenografted mice were treated with arnidiol. Mitochondrial fission and apoptosis were determined by immunofluorescence, flow cytometry and related molecular biological techniques. The interaction and colocalization of cofilin and Drp1 was determined by immunoprecipitation and immunofluorescence assays.

**Results:**

Arnidiol induces mitochondrial fission and apoptosis through mitochondrial translocation of Drp1 and cofilin. Importantly, the interaction of Drp1 and cofilin in mitochondria is involved in arnidiol-induced mitochondrial fission and apoptosis. Knockdown of either Drp1 or cofilin abrogated arnidiol-induced mitochondrial translocation, interaction of Drp1 and cofilin, mitochondrial fission and apoptosis. Only dephosphorylated Drp1 (Ser637) and cofilin (Ser3) were translocated to the mitochondria. Mutants of Drp1 S637A and cofilin S3A, which mimic the dephosphorylated forms, enhanced mitochondrial fission and apoptosis induced by arnidiol, whereas mutants of Drp1 S637D and cofilin S3E, which mimic the phosphorylated forms, suppressed mitochondrial fission and apoptosis induced by arnidiol. A mechanistic study revealed that ROCK1 activation plays an important role in the arnidiol-mediated Drp1 and cofilin dephosphorylation and mitochondrial translocation, mitochondrial fission, and apoptosis.

**Conclusions:**

Our data reveal a novel role of both Drp1 and cofilin in the regulation of mitochondrial fission and apoptosis and suggest that arnidiol could be developed as a potential agent for the treatment of human cancer.

## Background

Mitochondria are the main cellular organelles that undergo dynamic changes in response to physiological and pathological changes. These organelles are responsible for driving cell life and death through mitochondrial network structure homeostasis, which is determined by a balance of fission and fusion [1]. Mitochondrial fission is important for maintaining cellular functions, including cellular development and homeostasis, and apoptosis [2, 3]. The cytoplasmic dynamin-related GTPase Drp1 plays a key role in the regulation of mitochondrial fission. During apoptosis, Drp1 foci accumulate on mitochondria and mediate dramatic mitochondrial fission prior to cytochrome c release and caspase activation [4, 5]. Drp1 activity is rapidly regulated by the opposing effects of phosphorylation at two key serines. Phosphorylation of serine 616 increases Drp1 activity, whereas phosphorylation of serine 637 decreases it [6]. Each serine is targeted by different kinases and phosphatases, thereby linking mitochondrial fission to crucial cellular processes [7].

Cofilin, a member of the actin-depolymerizing factor (ADF) protein family, is best known as a regulator of actin filament nonequilibrium assembly and disassembly [8]. Cofilin has crucial roles in tumor progression, invasion, metastasis, and apoptosis [9–11]. It has recently been reported that cofilin functions as a regulator of mitochondrial apoptosis through regulating mitochondrial dynamics and functions [12]. During the induction of apoptosis, cofilin can translocate to mitochondria before cytochrome c release [13]. Cofilin activity is regulated by phosphorylation at serine 3. Dephosphorylation of Ser3 leads to cofilin activation [14]. The main protein phosphatases known to activate cofilin are serine/threonine phosphatases type 1 (PP1) and type 2A (PP2A), slingshot (SSH), and chronophin [14–16].

Rho-associated, coiled-coil containing protein kinases (ROCKs), the effectors of the Rho family of small GTPases, belong to a family of serine/threonine kinases [17]. The ROCK family contains two members: ROCK1 and ROCK2, which share 65% overall identity and 92% identity in the kinase domain [18]. Recent evidence has revealed that ROCK1 plays a critical role in regulating apoptosis in various cell types and animal models [19–21]. ROCK1-mediated apoptotic signaling may involve a mitochondrion-dependent intrinsic pathway [22]. It has recently been shown that ROCK1 plays an important role in regulating mitochondrial fission through the recruitment of Drp1 to the mitochondria [23]. ROCK1 has also been shown to be involved in the regulation of dephosphorylation and mitochondrial translocation of cofilin, leading to mitochondrial fission and apoptosis [24]. However, the detailed mechanism by which ROCK1 regulates mitochondrial fission and apoptosis by mediating the dephosphorylation and mitochondrial translocation of Drp1 and cofilin remains largely unknown.

**Fig. 1:**
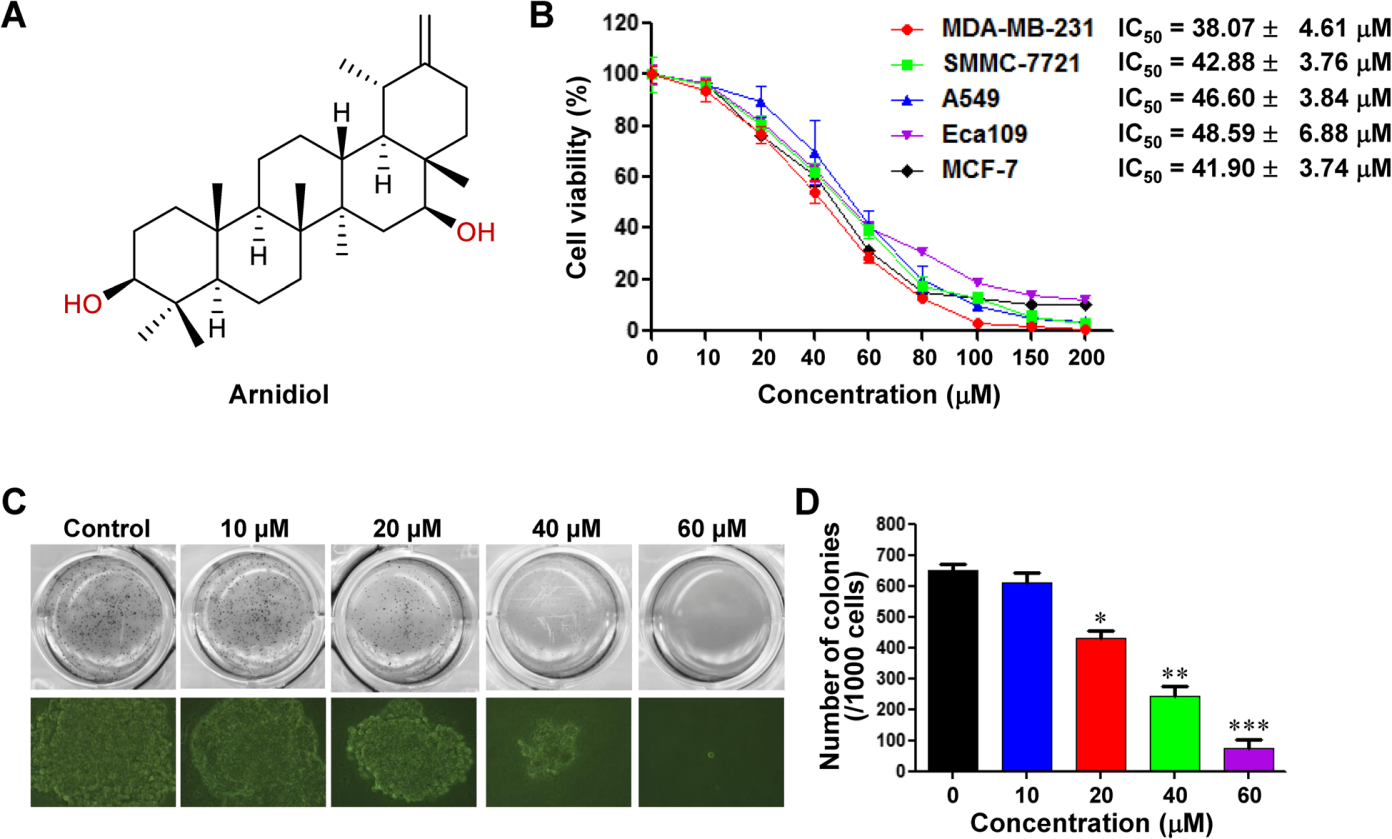
Arnidiol inhibits cell proliferation and colony formation in human cancer cells. **a** The chemical structure of Arnidiol (Arn). **b** Multiple cancer cell lines were treated with various doses of Arn for 48 h, and cell proliferation was measured by MTT assay. **c** and **d** Colony formation was detected using a soft agar assay in MDA-MB-231 cells (mean ± SD for 3 independent experiments, ^*^P < 0.05, ^**^P < 0.01 or ^***^P < 0.001 compared with control)

Arnidiol, taraxast-20(30)-ene-3b,16b-diol (Fig. 1a), is a pentacyclic triterpene diol isolated from *Tephroseris kirilowii* (Turcz.) Holub. Arnidiol has multiple pharmacological activities, including anti-inflammatory, antitubercular, chemopreventive, and cytotoxic activities [25–27]. The antitumor effects of arnidiol have recently attracted considerable attention. Arnidiol inhibits cell proliferation in various cancer cell lines, including leukemia (HL60), lung (A549), duodenal (AZ521), and breast (SK-BR-3) cancer cell lines [27, 28]. A recent study indicated that the taraxastane triterpenoid derivative induced typical apoptotic cell death in human leukemia HL60 cells [27]. However, the apoptotic activities of arnidiol in human cancer cells have not yet been explored, nor has the mechanism by which arnidiol induces apoptosis been examined in depth.

In the present study, we discovered that arnidiol inhibits cell proliferation in various cancer cell lines. For the first time, we showed that arnidiol selectively induces apoptosis in human cancer cells through induction of mitochondrial fission. Mechanistically, ROCK1 activation plays a critical role in arnidiol-mediated dephosphorylation of Drp1 (Ser637) and cofilin (Ser3), leading to their mitochondrial translocation, resulting in mitochondrial fission, and culminating in cytochrome c release and apoptosis. These findings provide a novel mechanistic basis for the application of arnidiol in the treatment of human cancer.

## Materials and methods

### Chemicals and antibodies

Arnidiol was isolated from *Tephroseris kirilowii* (Turcz.) Holub. Antibodies against C-Caspase 3 (9661S), phospho-Drp1 (S616, 3455), phospho-Drp1 (S637, 4876), and Drp1 (8570) were purchased from Cell Signaling Technology (Boston, MA, USA); GAPDH (AF0006) was purchased from Beyotime (Shanghai, China); COX4 (200147) and Cleaved-PARP (380374) were purchased from Zen-bio (Chengdu, China); PARP (1078–1) was purchased from Epitomics (Burlingame, USA); ROCK1 (ab45171), phospho-Cofilin (S3, ab12866) were purchased from Abcam (Cambridge, UK); PP2A (610555) was purchased from BD Biosciences (Franklin, NJ, USA). Cofilin (sc-376,476), Cytochrome. C (sc-13,156), Fis1 (sc-376,447), MFF (sc-398,617), Mfn1 (sc-166,644), Mfn2 (sc-515,647), OPA1 (sc-393,296), PP1 (sc-7482) were purchased from Santa Cruz Biotechnology (Dallas, TX, USA).

### Cell culture

MDA-MB-231 and MCF-7 breast cancer cells, A549 non-small cell lung cancer cells were obtained from the American Type Culture Collection (ATCC, Manassas, VA) and cultured in DMEM medium. SMMC-7721 hepatocellular carcinoma and Eca109 esophageal carcinoma cells were obtained from the Bena Culture Collection (Beijing, China) and cultured in RPMI1640 medium. All media comprised 10% fetal bovine serum (FBS). All cell lines were cultured at 37 °C in a humidified atmosphere with 5% CO_2_ in air.

### Cell viability (MTT) assay

Cells were seeded in 96 well plates (3.5 × 10^3^/well) and treated as indicated experimental conditions for 48 h. 20 μl MTT (5 mg/ml) was added in each well and incubated at 37 °C for 4 h. Each well was supplemented with 150 μl DMSO to dissolve the formazan. The absorbance was measured at 490 nm using microplate reader. The cell viabilities were normalized to the control group.

### Soft agar assay

Sustainment gel was mixed with 0.6% agarose (Sigma-Aldrich) in a cell culture medium in 12 well plates. 1000 cells were cultured in cultivate gel above concretionary sustainment gel (mixed with 0.3% agarose in cell culture medium with 10% FBS). After 30 days, the colonies were photographed by using Microscope (Jiangsu, China), then, 100 μl MTT (5 mg/ml) was added in each well and incubated at 37 °C for 0.5–1 h and scanned with MICROTEK Scan Marker (Shanghai, China).

### Apoptosis assay

Cells were stained with annexin V-FITC and PI to evaluate apoptosis by flow cytometry according to the manufacturer’s instructions (BD Biosciences PharMingen). Briefly, 1 × 10^6^ cells were washed twice with PBS and stained with 5 μl of PI (50 μg/ml) and 2 μl of Annexin V-FITC in 1× binding buffer for 15 min at room temperature in the dark. Quantification of apoptotic cells was performed by flow cytometry using a FACScan cytofluorometer (BD Biosciences). Both early and late apoptotic cells were included in the cell death determinations.

### Mitochondrial and cytosolic fractionation

Mitochondrial and cytosolic fractions were obtained as previously described [29]. Cell pellets were washed twice with PBS and resuspended in 5x Buffer A (10 mM KCl, 20 mM HEPES, 1.5 mM MgCl_2_, 1 mM EGTA, 1 mM EDTA, 2 mM leupeptin, 1 mM Na_3_VO_4_, 1 mM PMSF, 1 mM DTT, 2 mM pepstatin and 250 mM sucrose). Cells were homogenized by passing 15 times through a 22-gauge needle. The homogenate was centrifuged at 1000 g at 4 °C for 10 min, then transfer the supernatant continue centrifuged at 3500 g at 4 °C for 10 min, The pellet fraction was considered the “mitochondrial” fraction. The supernatant fraction was centrifuged at 12000 g at 4 °C for 10 min, the supernatant fraction was considered the “cytosolic” fraction.

### Western blots and immunoprecipitation

The protein samples (30–50 μg) were separated using SDS-PAGE and transferred to PVDF membranes (Bio-Rad, 162–0177). After blocking with 5% fat-free dry milk in 1 × Tris-buffered saline (TBS), the membrane was probed overnight with primary antibodies at 4 °C. Protein bands were detected by incubating with horseradish peroxidase-conjugated antibodies (Kirkegaard and Perry Laboratories, Gaithersburg, MD, USA) and visualized with enhanced chemiluminescence reagent (Perkin-Elmer, Boston, MA, USA). For immunoprecipitation analysis, equal quantities of proteins were incubated with primary antibodies at 4 °C on a rocking platform. Immune complexes were collected with protein A/G agarose beads (Beyotime Technology), washed in PBS five times, and subjected to Western blot.

### Immunofluorescence

Cells were seeded on coverslips and cultured in 24 well plates for 24 h, cells were treated with drugs for 48 h. The mitochondria were stained with MitoTracker Deep Red FM (Molecular Probes, Carlsbad, USA) according to the manufacturer’s instructions. Cells were fixed with 4% formaldehyde (Beyotime Biotechnology) for 30 min, permeabilized with 0.1% Triton X-100 in PBS for 7 min, then blocked with goat serum (Beyotime Biotechnology) in PBS for 30 min. The cells were incubated overnight with primary antibodies at 4 °C, followed by the appropriate secondary antibodies at 37 °C for 1 h. The cells were viewed using a laser-scanning confocal microscope (Zeiss, Germany). All images were analyzed by ImageJ software (MD, USA).

### RNA interference and site mutant

The target sequence of cofilin shRNA (5′-CCGGAAGGTGTTCAATGACATGAAACTCGAGTTTCATGTCATTGAACACCTTTTTTTG-3′) and ROCK1 shRNA (5′-CCGGGCACCAGTTGTACCCGATTTACTCGAGTAAATCGGGTACAACTGGTGCTTTTTG − 3′) was constructed by Gene Chem Co. Ltd. (Shanghai, China). Drp1 shRNA plasmid (sc-43,732) and control shRNA plasmid (sc-108,060) were purchased from Santa Cruz Biotechnology. Plasmids were co-transfected with pLP1, pLP2 and pLP/VSVG (Invitrogen) into 293FT cells using Lipofectamine 3000 (Invitrogen) according to the manufacturer’s instructions. The supernatant containing the lentivirus was harvested and infected with MDA-MB-231 cells. Cells were subsequently selected with 8 μg/mL puromycin to establish stable cell lines. Site mutant plasmids of cofilin (Cofilin^S3A^ and Cofilin^S3E^) were a gift from Professor James Bamburg (Colorado State University, USA). Site mutant plasmids of Drp1 (Drp1^S637D^ and Drp1^S637A^) were generated using the QuickChange Site-Directed Mutagenesis Kit (Stratagene, CA, USA) with the following primers:

S637D (FW: 5′-GCACGAAAACTAGATGCTCGGGAACAG-3′;

RV: 5′-CTGTTCCCGAGCATCTAGTTTTCGTGC-3′),

S637A (FW:5′-GCACGAAAACTAGCTGCTCGGGAACAG-3′;

RV: 5′-CTGTTCCCGAGCAGCTAGTTTTCGTGC-3′).

MDA-MB-231 were transfected with plasmids using Lipofectamine 3000 according to the manufacturer’s instructions.

### Xenograft assay

Female nude mice (5–6 weeks old) were purchased from Vital River Laboratories (VRL, Beijing, China) and fed a standard animal diet and water. The animal studies were approved by the University Institutional Animal Care and Use Committee. MDA-MB-231 cells were suspended in a 1:1 ratio in DMEM medium with a Matrigel basement membrane matrix (Sigma, E1270). Cells (4 × 10^7^) were inoculated in the right legs of mice. After tumor inoculation, the mice were randomly assigned into 3 treatment groups (16 mice per group, 6 mice were used for body weight and tumor volume measurement, the others were used for survival analysis). The mice were treated with Arnidiol (40 mg/kg, 80 mg/kg) or an equal volume of vehicle by intraperitoneal injection. The body weight and tumor diameters were measured every 5 days. The mice were euthanized 30 d after medication. The tumors were excised and were either formalin-fixed or flash-frozen at − 20 °C. H&E, TUNEL, and immunohistochemical analyses were performed as previously described [30].

### Statistical analysis

All data values are represented as mean ± SD. The comparisons were performed using Student’s t-test or one-way analysis of variance (ANOVA). Survival analysis in vivo was performed using the Kaplan–Meier method and significance was calculated using the log-rank test. ^*^P < 0.05, ^**^P < 0.01, and ^***^P < 0.001 were regarded as significant differences.

## Results

### Arnidiol inhibits cell proliferation and colony formation and induces apoptosis in human cancer cells

To evaluate the effects of arnidiol on the growth of human cancer cells, the cell viabilities of a variety of human cancer cells, including MDA-MB-231 and MCF-7 breast cancer cells, SMMC-7721 hepatocellular carcinoma cells, A549 non-small-cell lung cancer cells, and Eca109 esophageal carcinoma cells, were determined by MTT assay. We found that the cell viabilities were decreased in a dose-dependent manner in these cancer cells treated with arnidiol (Fig. 1b). We also examined the effects of arnidiol on colony formation in MDA-MB-231 cells in vitro by using a soft agar assay. As shown in Fig. 1c and d, treating MDA-MB-231 cells with arnidiol significantly decreased the number of colonies in a dose-dependent manner. These results indicate that arnidiol could inhibit cell proliferation and tumorigenesis in human cancer cells.

**Fig. 2:**
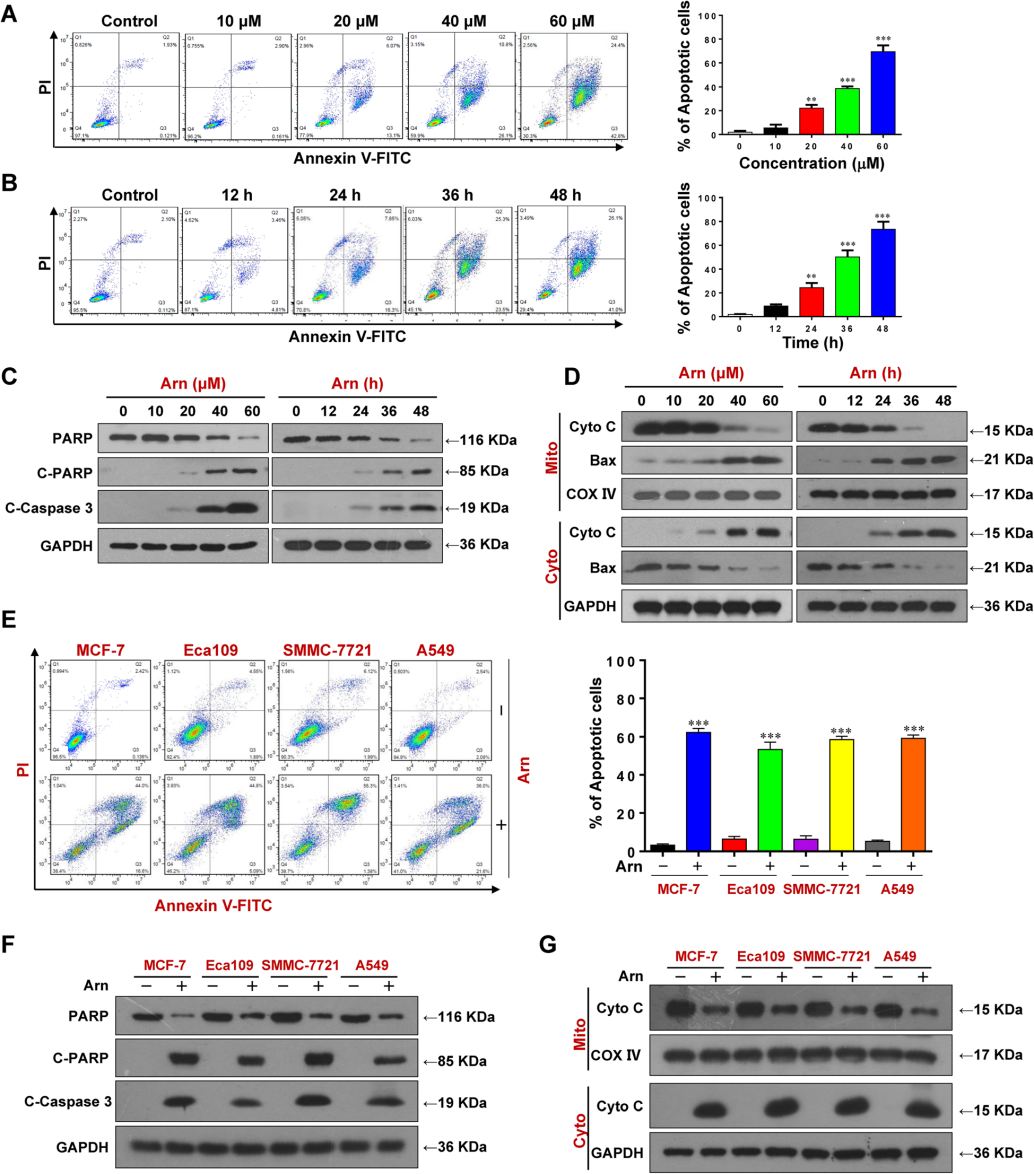
Arnidiol induces apoptosis in human breast cancer cells. For A-D, MDA-MB-231 cells were treated with various concentrations of Arn for 48 h or with Arn (60 μM) for different time intervals as indicated. **a** and **b** Apoptosis was determined by Annexin V-FITC/PI staining and flow cytometry (mean ± SD for 3 independent experiments; ^**^P < 0.01 or ^***^P < 0.001 compared with control). **c** and **d** The total cellular extract, cytosol and mitochondrial fractions were prepared and subjected to western blot using antibodies against total PRAP, cleaved PARP (C-PARP), cleaved caspase-3 (C-Caspase-3), cytochrome c (Cyto C) and Bax. GAPDH and COX IV were used as loading controls. For E-G, MCF-7, Eca109, SMMC-7721 and A549 cells were treated with Arn (60 μM) for 48 h. **e** Apoptosis was determined by Annexin V-FITC/PI staining and flow cytometry (mean ± SD for 3 independent experiments; ^***^P < 0.001 compared with control). **f** and **g** The total cellular extract, cytosol and mitochondrial fractions were prepared and subjected to western blot using antibodies against total PRAP, cleaved PARP (C-PARP), cleaved caspase-3 (C-Caspase-3) and cytochrome c (Cyto C). GAPDH and COX IV were used as loading controls

We next investigated the effects of arnidiol on apoptosis in MDA-MB-231 cells. Treatment of cells with arnidiol resulted in a pronounced increase in apoptosis in MDA-MB-231 cells in a dose- and time-dependent manner (Fig. 2a and b). Consistent with these findings, arnidiol treatment caused degradation of PARP and cleavage/activation of caspase-3 (Fig. 2c). Bax, a pro-apoptotic member of the Bcl-2 family of proteins, has the ability to form transmembrane pores large enough to allow cytochrome c release [31]. It has been proposed that mitochondrial translocation of Bax causes cytochrome c release from mitochondria, leading to apoptosis [32]. We then examined the effects of arnidiol on mitochondrial translocation of Bax and release of cytochrome c. Treating cells with arnidiol resulted in release of cytochrome c from the mitochondria into the cytosolic fraction and mitochondrial translocation of Bax in a dose- and time-dependent manner (Fig. 2d). Induction of apoptosis was also observed in MCF-7, Eca109, SMMC-7721 and A549 cells treated with arnidiol (Fig. 2e-g). These findings suggest that arnidiol induces mitochondrial injury and apoptosis in human cancer cells.

### Arnidiol induces mitochondrial fission in human cancer cells

**Fig. 3:**
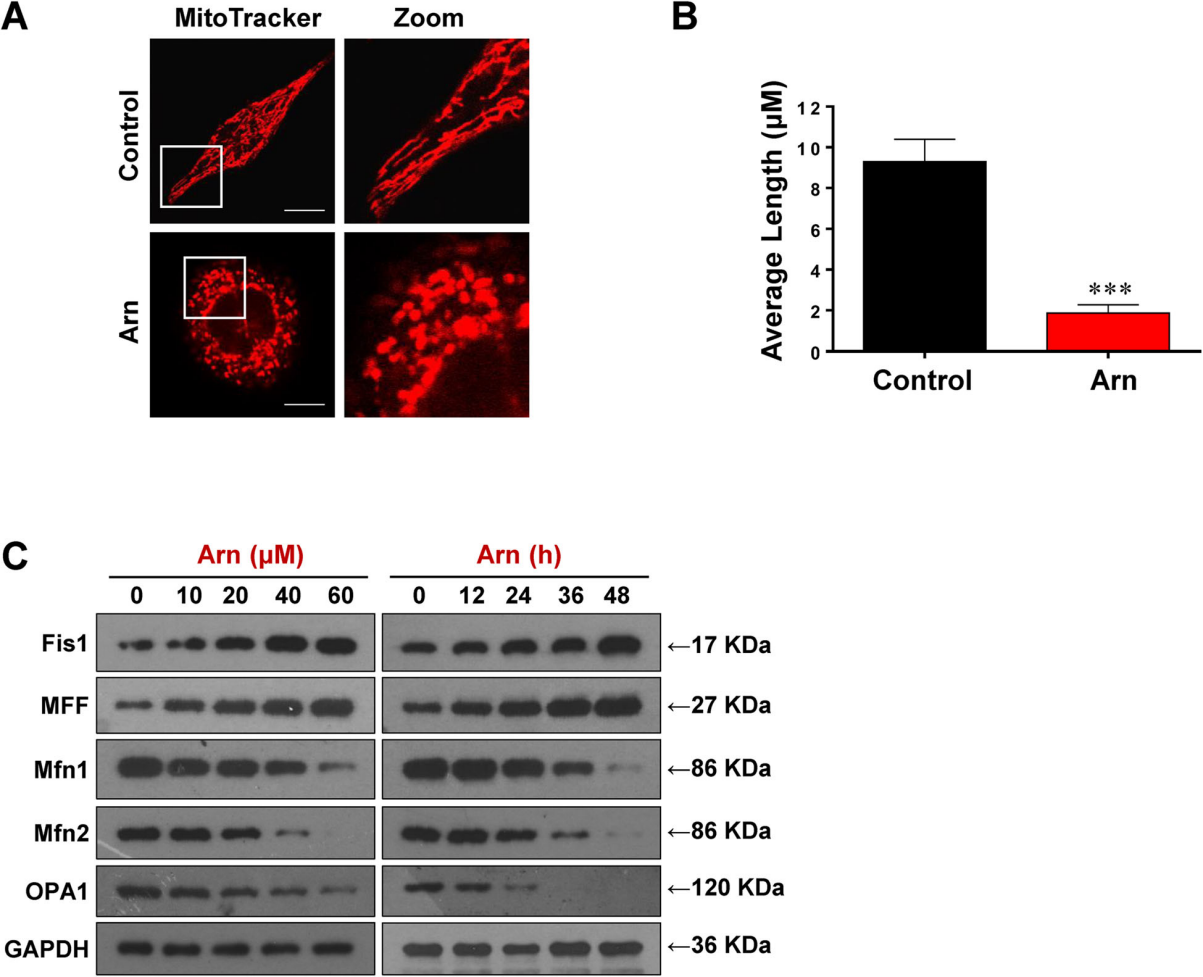
Arnidiol induces mitochondrial fission in human breast cancer cells. **a** and **b** MDA-MB-231 cells were treated with Arn (60 μM) for 48 h, mitochondrial morphology was observed by MitoTracker Red CMXRos staining and confocal microscopy. Scale bars: 10 μm. Mitochondrial length was measured with ImageJ software. 50 cells from 3 independent experiments (mean ± SD, ^***^P < 0.001 compared with control). **c** MDA-MB-231 cells were treated with various concentrations of Arn for 48 h or with Arn (60 μM) for different time intervals as indicated, the total cellular extracts were prepared and subjected to western blot using antibodies against Fis1, MFF, Mfn1, Mfn2 and OPA1. GAPDH was used as loading control

Increasing evidence supports that mitochondrial fission participates in Bax-mediated permeabilization of the outer mitochondrial membrane and cytochrome c release, leading to mitochondrial apoptosis [33]. We next examined the effects of arnidiol on mitochondrial morphology using MitoTracker Red CMXRos. Notably, treatment with arnidiol resulted in a significant increase in the proportion of cells with fragmented mitochondria compared to control cells that exhibited filamentous mitochondria (Fig. 3a and b).

Recent studies revealed that a number of components of fission proteins, including fission protein 1 (Fis1) and mitochondrial fission factor (MFF), and fusion proteins, including mitofusin 1 and 2 (Mfn1 and Mfn2), and optic atrophy 1 (OPA1), play important roles in the regulation of mitochondrial fission [34–36]. To evaluate the molecular mechanism by which arnidiol induces mitochondrial fragmentation in human breast cancer cells, we examined the effects of arnidiol on the expression of these fission- and fusion-related proteins. Western blot analysis revealed that arnidiol treatment significantly increased the expression of Fis1 and Mff and decreased the expression of OPA1 and Mfn1/2 in a dose- and time-dependent manner (Fig. 3c). These results suggest that arnidiol induces mitochondrial fission, leading to apoptosis.

### Mitochondrial translocation and interaction of Drp1 and cofilin are required for arnidiol-induced mitochondrial fission

**Fig. 4:**
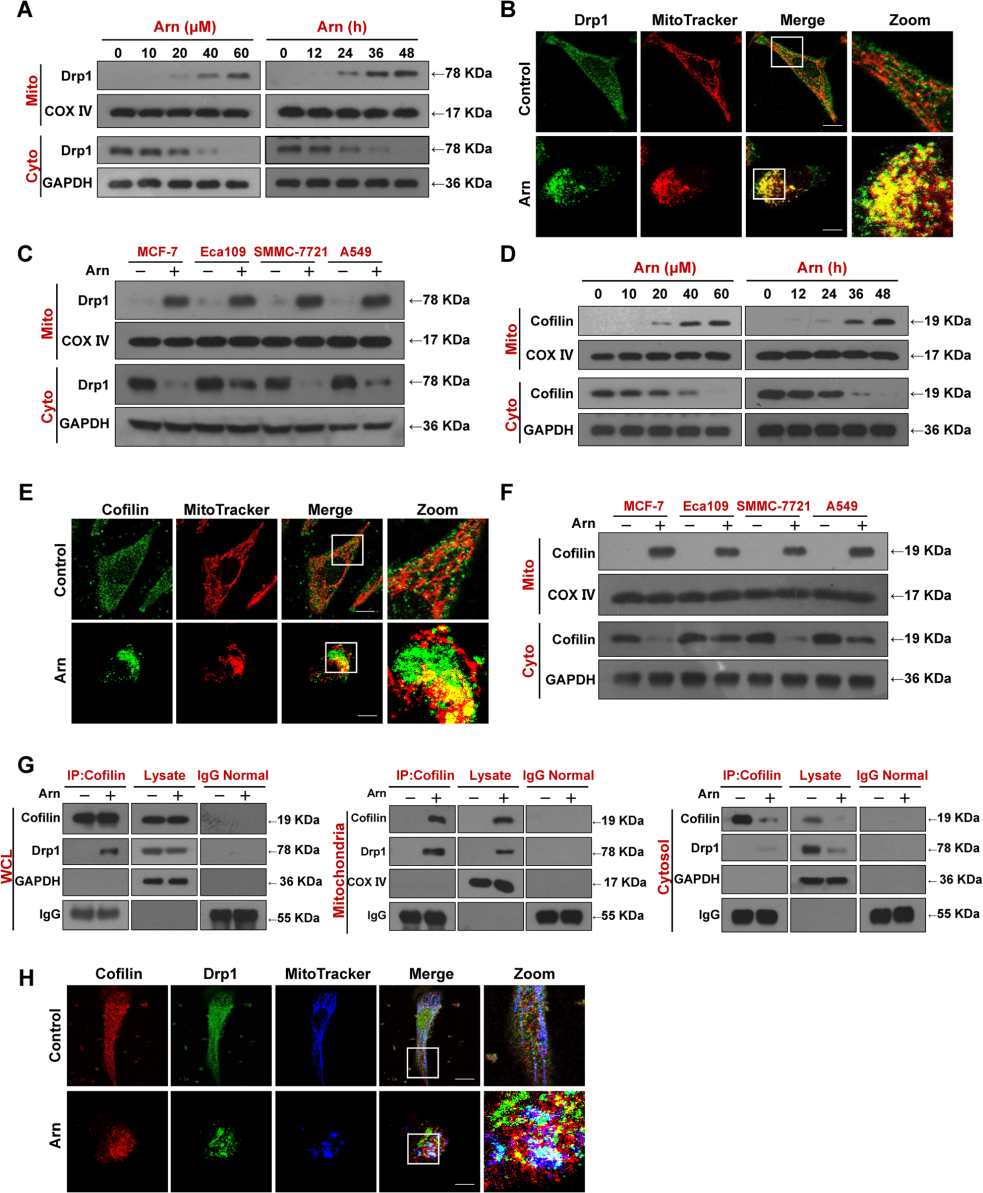
Arnidiol induces mitochondrial translocation of Drp1 and Cofilin. **a** and **d** MDA-MB-231 cells were treated with various concentrations of Arn for 48 h or with Arn (60 μM) for different time intervals as indicated, cytosol and mitochondrial fractions were prepared and subjected to western blot using antibodies against Drp1 and Cofilin. GAPDH and COX IV were used as loading controls. **b** and **e** MDA-MB-231 cells were treated with Arn (60 μM) for 48 h, the colocalization of MitoTracker (red) and Drp1 (green) or Cofilin (green) was examined using confocal microscopy. Scale bars: 10 μm. **c** and **f** MCF-7, Eca109, SMMC-7721 and A549 cells were treated with Arn (60 μM) for 48 h, cytosol and mitochondrial fractions were prepared and subjected to western blot using antibodies against Drp1 and Cofilin. GAPDH and COX IV were used as loading controls. **g** MDA-MB-231 cells were treated with Arn (60 μM) for 48 h, WCL, mitochondrial and cytosol fractions were prepared and subjected to immunoprecipitation using anti-Cofilin, the associated Cofilin and Drp1 were determined using immunoblotting. **h** MDA-MB-231 cells were treated with Arn (60 μM) for 48 h, the colocalization of Cofilin (red), Drp1 (green), and MitoTracker (blue) was examined using confocal microscopy. Scale bars: 10 μm

Dynamin-related protein 1 (Drp1), a member of the dynamin family of GTPases, is the key component of the mitochondrial fission machinery [37]. A number of studies have revealed that mitochondrial translocation of Drp1 is a prerequisite for the induction of mitochondrial fission and apoptosis. We next examined whether mitochondrial translocation of Drp1 is necessary for arnidiol to induce mitochondrial fission. Treatment of cells with arnidiol significantly increased the levels of Drp1 in mitochondria and decreased Drp1 levels in the cytosol in a dose- and time-dependent manner (Fig. 4a). We also detected the subcellular localization of Drp1 in response to arnidiol treatment by using immunofluorescence microscopy. As shown in Fig. 4b, Drp1 signals were localized at the mitochondria after arnidiol treatment. Interestingly, mitochondrial fission was also observed following arnidiol treatment. Mitochondrial translocation of Drp1 was also observed in MCF-7, Eca109, SMMC-7721 and A549 cells treated with arnidiol (Fig. 4c). These findings suggest that mitochondrial translocation of Drp1 is required for arnidiol-mediated mitochondrial fission.

Recent evidence reveals that cofilin, a key regulator of actin dynamics, has a critical role in regulating mitochondrial function and shape [38]. It has recently been reported that mitochondrial translocation of cofilin is associated with mitochondrial fission and mitochondrial membrane permeabilization [12]. We next examined whether arnidiol affects the mitochondrial translocation of cofilin. Treatment of cells with arnidiol significantly increased the levels of cofilin in mitochondria and decreased the levels of cofilin in the cytosol in a dose- and time-dependent manner (Fig. 4d). Interestingly, immunofluorescence assays showed that cofilin signals were localized to the fragmented mitochondria of cells treated with arnidiol, whereas cofilin signals were not localized to the normal filamentous mitochondria of control cells (Fig. 4e). Mitochondrial translocation of cofilin was also observed in MCF-7, Eca109, SMMC-7721 and A549 cells treated with arnidiol (Fig. 4f). These results suggest that mitochondrial translocation of cofilin is also required for arnidiol-induced mitochondrial fission and apoptosis.

Since our data showed that mitochondrial translocation of both Drp1 and cofilin was required for arnidiol-induced mitochondrial fission, we questioned whether cofilin could interact with Drp1 in mitochondria during arnidiol treatment. Immunoprecipitation assays indicated that Drp1 was coimmunoprecipitated with cofilin in either whole cell lysates or mitochondria, whereas Drp1 was not coimmunoprecipitated with cofilin in the cytosolic fraction when cells were treated with arnidiol (Fig. 4g). Immunofluorescence assays showed that the colocalization of Drp1 and cofilin in mitochondria was observed in cells treated with arnidiol (Fig. 4h).

**Fig. 5:**
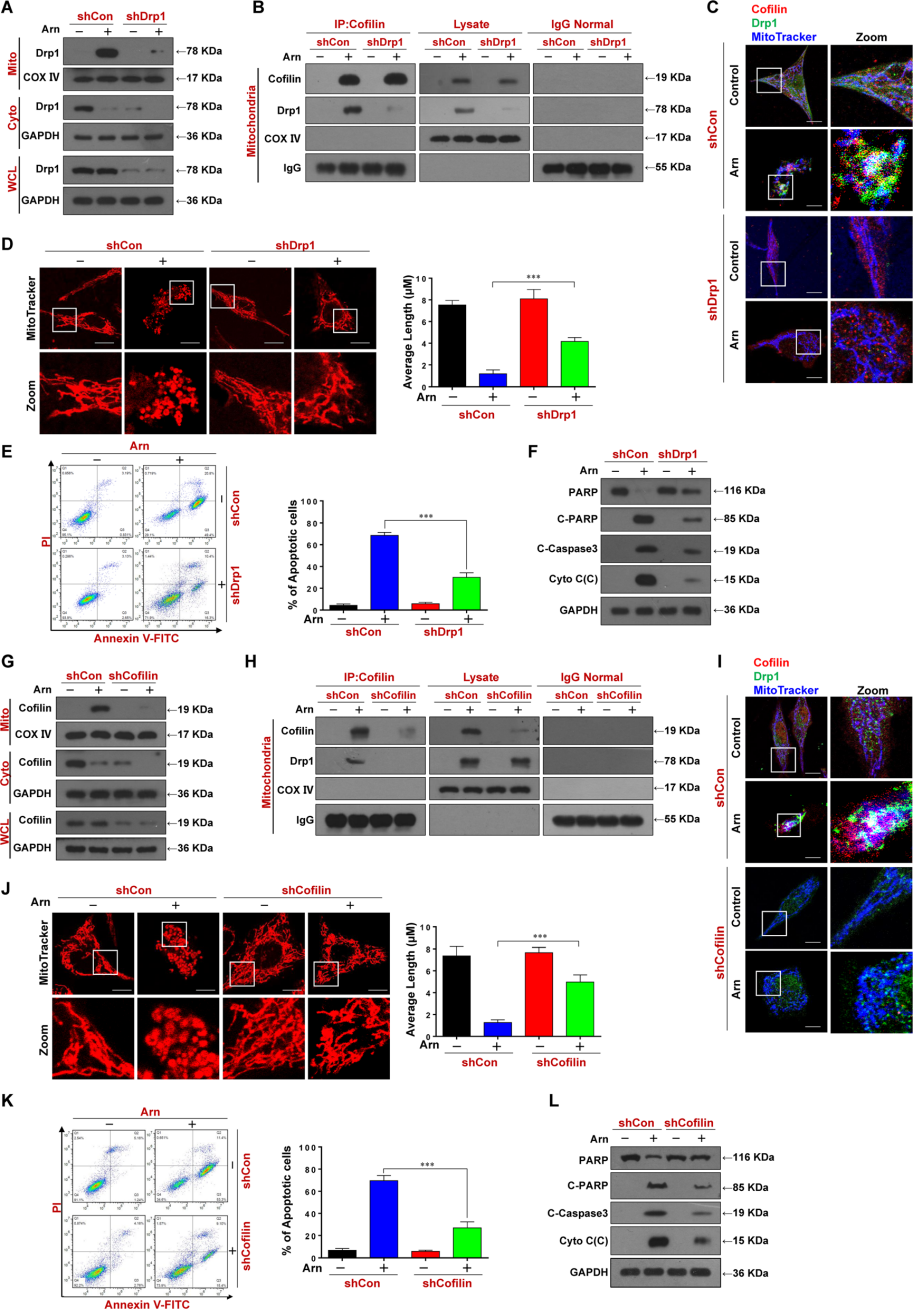
Drp1 or cofilin knockdown attenuates Arnidiol-mediated mitochondrial fission and apoptosis. For **a-f**, cells stably expressing shControl or shDrp1 were treated with Arn (60 μM) for 48 h. **a** WCL, cytosol and mitochondrial fractions were prepared and subjected to western blot using antibody against Drp1. **b** Mitochondrial fractions were prepared and subjected to immunoprecipitation using anti-Cofilin, the associated Cofilin and Drp1 were determined using immunoblotting. **c** The colocalization of Cofilin (red), Drp1 (green), and MitoTracker (blue) was examined using confocal microscopy. Scale bars: 10 μm. **d** Mitochondrial morphology was observed by MitoTracker Red CMXRos staining and confocal microscopy. Scale bars: 10 μm. Mitochondrial length was measured with ImageJ software. 50 cells of 3 independent experiments (mean ± SD, ^***^P < 0.001). **e** Apoptosis was detected by flow cytometry analysis (mean ± SD for 3 separate experiments, ^***^P < 0.001). **f** WCL, cytosol fractions were prepared and subjected to western blot using antibodies against total PRAP, C-PARP, C-Caspase-3 and Cyto C. GAPDH was used as loading control. For **g-l**, cells stably expressing shControl or shCofilin were treated with Arn (60 μM) for 48 h. **g** WCL, cytosol and mitochondrial fractions were prepared and subjected to western blot using antibody against Cofilin. **h** Mitochondrial fraction was prepared and subjected to immunoprecipitation using anti-Cofilin, the associated Cofilin and Drp1 were determined using immunoblotting. **i** The colocalization of Cofilin (red), Drp1 (green), and MitoTracker (blue) was examined using confocal microscopy. Scale bars: 10 μm. **j** Mitochondrial morphology was observed by MitoTracker Red CMXRos staining and confocal microscopy. Scale bars: 10 μm. Mitochondrial length was measured with ImageJ software. 50 cells of 3 independent experiments (mean ± SD, ^***^P < 0.001). **k** Apoptosis was detected by flow cytometry analysis (mean ± SD for 3 separate experiments, ^***^P < 0.001). **l** WCL, cytosol fractions were prepared and subjected to western blot using antibodies against total PRAP, C-PARP, C-Caspase-3 and CytoC. GAPDH was used as loading control

To further address the functional role of Drp1 in arnidiol-induced mitochondrial fission and apoptosis, a lentiviral shRNA approach was used to stably knockdown Drp1 expression. Knockdown of Drp1 efficiently attenuated arnidiol-mediated mitochondrial translocation of Drp1 (Fig. 5a). Knockdown of Drp1 also attenuated the interaction and colocalization of Drp1 and cofilin in the mitochondria (Fig. 5b and c). Furthermore, knockdown of Drp1 abrogated arnidiol-induced mitochondrial fission and apoptosis (Fig. 5d-f).

We also knocked down cofilin with a lentiviral shRNA to evaluate the functional role of cofilin in arnidiol-induced mitochondrial fission and apoptosis. Similar to the functional role of Drp1, knockdown of cofilin markedly reduced arnidiol-mediated mitochondrial translocation of cofilin (Fig. 5g). Knockdown of cofilin also attenuated the interaction and colocalization of Drp1 and cofilin in the mitochondria (Fig. 5h and i). Furthermore, knockdown of cofilin abrogated arnidiol-induced mitochondrial fission and apoptosis (Fig. 5j-l). Together, these findings suggest that the mitochondrial translocation and interaction of Drp1 and cofilin are essential for arnidiol-induced mitochondrial fission and apoptosis.

### Dephosphorylation of Drp1 (Ser637) and cofilin (Ser3) is required for arnidiol-induced mitochondrial fission and apoptosis

**Fig. 6:**
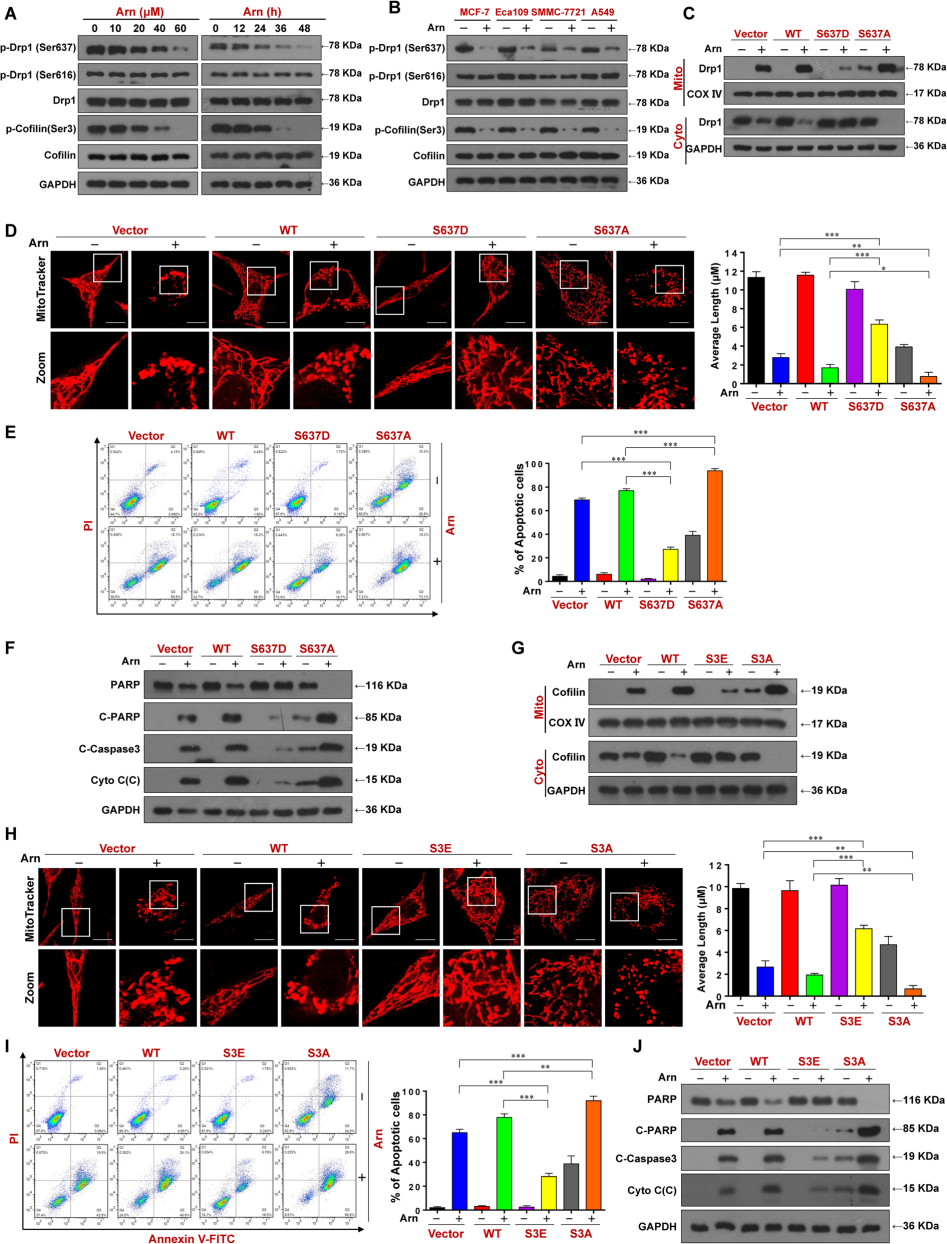
Dephosphorylation of Drp1 (Ser637) and cofilin (Ser3) is required for arnidiol-induced mitochondrial fission and apoptosis. **a** MDA-MB-231 cells were treated with various concentrations of Arn for 48 h or with Arn (60 μM) for different time intervals as indicated, WCL were prepared and subjected to Western blot analysis using antibodies against p-Drp1 (S637), p-Drp1 (S616), Drp1, p-Cofilin (S3) and Cofilin. GAPDH was used as loading control. **b** MCF-7, Eca109, SMMC-7721 and A549 cells were treated with Arn (60 μM) for 48 h, WCL were prepared and subjected to Western blot analysis using antibodies against p-Drp1 (S637), p-Drp1 (S616), Drp1, p-Cofilin (S3) and Cofilin. GAPDH was used as loading control. For **c-f**, MDA-MB-231 cells were transfected with vector control or Drp1^WT^ or Drp1^S637D^ or Drp1^S637A^ were treated with Arn (60 μM) for 48 h. **c** Mitochondrial and cytosol fractions were prepared and subjected to Western blot analysis using antibodies against Drp1, GAPDH and COX IV were used as loading controls. **d** Mitochondrial morphology was observed by MitoTracker Red CMXRos staining and confocal microscopy. Scale bars: 10 μm. Mitochondrial length was measured with ImageJ software. 50 cells of 3 independent experiments (mean ± SD, ^*^P < 0.05, ^**^P < 0.01 or ^***^P < 0.001). **e** Apoptosis was detected by flow cytometry analysis (mean ± SD for 3 separate experiments, ^***^P < 0.001). **f** WCL and cytosol fractions were prepared and subjected to western blot using antibodies against total PRAP, C-PARP, C-Caspase 3 and Cyto C. GAPDH was used as loading control. For **g-j**, MDA-MB-231 cells were transfected with vector control or Cofilin^WT^ or Cofilin^S3E^ or Cofilin^S3D^ and treated with Arn (60 μM) for 48 h. **g** Mitochondrial and cytosol fractions were prepared and subjected to Western blot analysis using antibodies against Cofilin, GAPDH and COX IV were used as loading controls. **h** Mitochondrial morphology was observed by MitoTracker Red CMXRos staining and confocal microscopy. Scale bars: 10 μm. Mitochondrial length was measured with ImageJ software. 50 cells of 3 independent experiments (mean ± SD, ^**^P < 0.01 or ^***^P < 0.001). **i** Apoptosis was detected by flow cytometry analysis (mean ± SD for 3 separate experiments, ^**^P < 0.01 or ^***^P < 0.001). **j** WCL and cytosol fractions were prepared and subjected to western blot using antibodies against total PRAP, C-PARP, C-Caspase-3 and Cyto C. GAPDH was used as loading control

It has been shown that the phosphorylation status of Drp1 and cofilin can influence their ability to translocate to mitochondria and induce mitochondrial fission [7, 13]. Recent studies revealed that only dephosphorylated Drp1 (Ser637) and cofilin (Ser3) are translocated to mitochondria during the initiation of apoptosis [6, 13]. We next investigated whether arnidiol could affect the phosphorylation status of Drp1 and cofilin. Exposure of cells to arnidiol resulted in decreases in the levels of phospho-Drp1 (Ser637) and phospho-cofilin (Ser3) in a dose- and time-dependent manner. In contrast, phosphorylation of Drp1 (Ser616) was not changed in cells treated with arnidiol (Fig. 6a). Dephosphorylation of Drp1 (Ser637) and cofilin (Ser3) was also observed in MCF-7, Eca109, SMMC-7721 and A549 cells treated with arnidiol (Fig. 6b).

To further determine whether the phosphorylation status of Drp1 and cofilin could influence their ability to translocate to mitochondria and induce apoptosis, mutants of Drp1 Ser637 (S637A) and cofilin Ser3 (S3A) that mimic the dephosphorylated forms and mutants of Drp1 Ser637 (S637D) and cofilin Ser3 (S3E) that mimic the phosphorylated forms were generated. Interestingly, overexpression of Drp1 S637A enhanced the mitochondrial translocation of Drp1 in arnidiol-treated cells. In contrast, overexpression of Drp1 S637D reduced mitochondrial accumulation of Drp1 in arnidiol-treated cells (Fig. 6c). Overexpression of Drp1 S637A increased mitochondrial fission mediated by arnidiol, whereas Drp1 S637D reduced this effect (Fig. 6d). Furthermore, Drp1 S637A increased PARP degradation, caspase 3 activation, cytochrome c release, and apoptosis in arnidiol-treated cells, whereas Drp1 S637D reduced these effects (Fig. 6e and f).

Similarly, overexpression of cofilin S3A enhanced mitochondrial translocation of cofilin, mitochondrial fission, and apoptosis in arnidiol-treated cells, whereas cofilin S3E reduced these effects (Fig. 6g-j). Taken together, these findings indicate that the dephosphorylation of both Drp1 (S637) and cofilin (Ser3) is required for the mitochondrial translocation of Drp1 and cofilin and for their abilities to induce mitochondrial fission and apoptosis mediated by arnidiol.

### Activation of ROCK1 is involved in the arnidiol-mediated dephosphorylation and mitochondrial translocation of Drp1 and cofilin and in mitochondrial fission and apoptosis

**Fig. 7:**
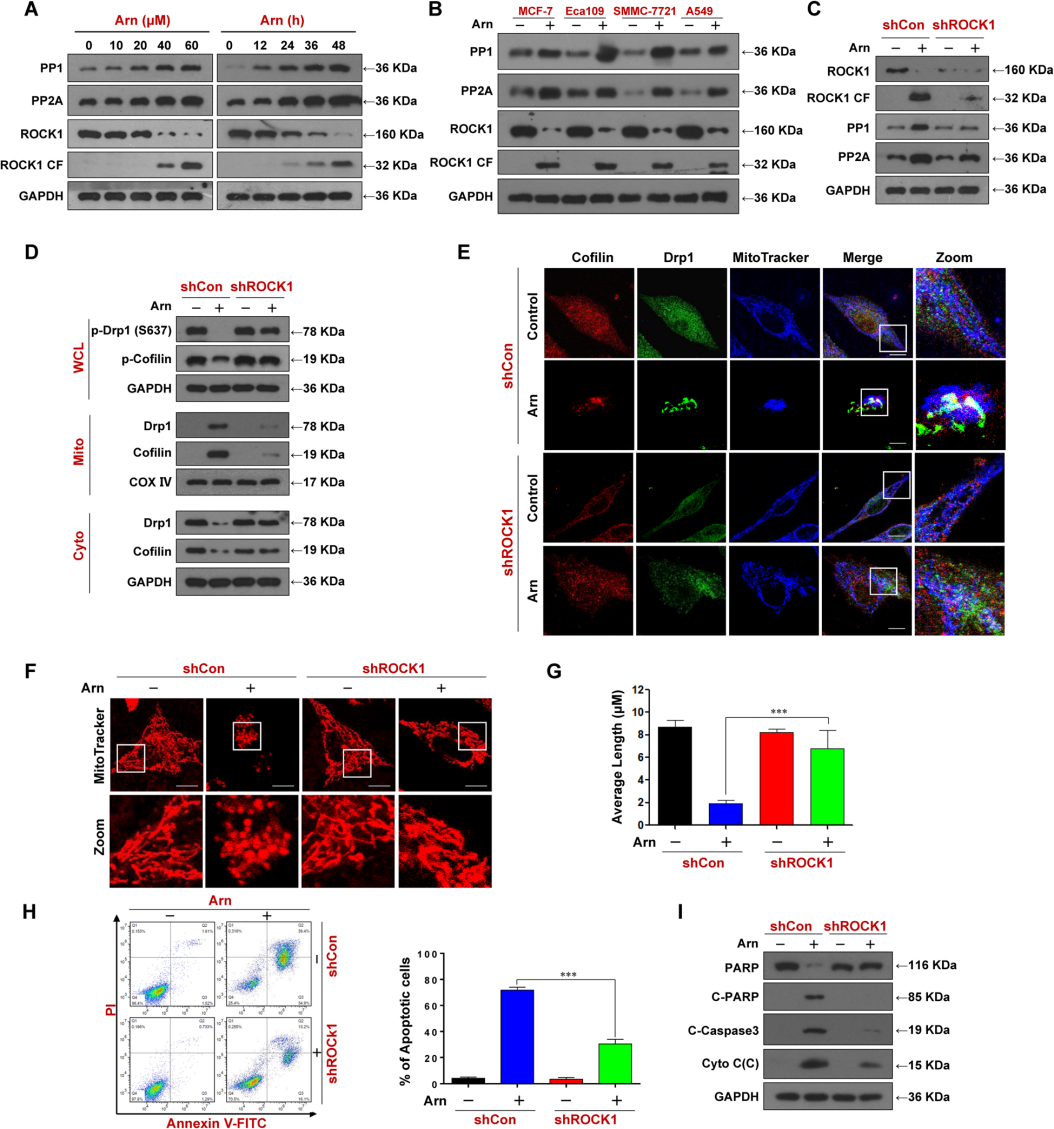
ROCK1 activation is involved in arnidiol-mediated dephosphorylation and mitochondrial translocation of Drp1 and cofilin, mitochondrial fission and apoptosis. **a** MDA-MB-231 cells were treated with various concentrations of Arn for 48 h or with Arn (60 μM) for different time intervals as indicated, WCL were prepared and subjected to western blot using antibodies against PP1, PP2A and ROCK1. GAPDH was used as loading control. **b** MCF-7, Eca109, SMMC-7721 and A549 cells were treated with Arn (60 μM) for 48 h, WCL were prepared and subjected to Western blot analysis using antibodies against PP1, PP2A and ROCK1. GAPDH was used as loading control. For **c-i**, cells stably expressing shControl or shROCK1 were treated with Arn (60 μM) for 48 h. **c** and **d** WCL, cytosol and mitochondrial fractions were prepared and subjected to western blot using antibodies against ROCK1, PP1, PP2A, p-Drp1 (S637), p-Cofilin, Drp1 and Cofilin, GAPDH and COX IV were used as loading controls. **e** The colocalization of Cofilin (red), Drp1 (green), and MitoTracker (blue) was examined using confocal microscopy. Scale bars: 10 μm. **f** Mitochondrial morphology was observed by MitoTracker Red CMXRos staining and confocal microscopy. Scale bars: 10 μm. **g** Mitochondrial length was measured with ImageJ software. 50 cells of 3 independent experiments (mean ± SD, ^***^P < 0.001). **h** Apoptosis was detected by flow cytometry analysis (mean ± SD for 3 separate experiments, ^***^P < 0.001). **i** WCL, cytosol fractions were prepared and subjected to western blot using antibodies against total PRAP, C-PARP, C-Caspase-3 and Cyto C. GAPDH was used as loading control

As the dephosphorylation of Drp1 and cofilin is regulated by the phosphatases PP1 and PP2A, which are regulated by the ROCK1 signaling pathway [39, 40], we next examined whether arnidiol could affect the expression of PP1, PP2A, and ROCK1. Exposure of cells to arnidiol decreased the levels of total ROCK1 and increased the cleavage of ROCK1 in a dose- and time-dependent manner (Fig. 7a). Treatment of cells with arnidiol also increased the expression of PP1 and PP2A in a dose- and time-dependent manner (Fig. 7a). Activation of ROCK1 and increased expression of PP1 and PP2A were also observed in MCF-7, Eca109, SMMC-7721 and A549 cells treated with arnidiol (Fig. 7b).

To further assess the functional role of ROCK1 activation in the regulation of mitochondrial fission and apoptosis through dephosphorylation and mitochondrial translocation of Drp1 and cofilin, a lentiviral shRNA approach was employed to stably knockdown ROCK1 expression (Fig. 7c). Knockdown of ROCK1 attenuated arnidiol-induced expression of PP1 and PP2A (Fig. 7c). Knockdown of ROCK1 also attenuated arnidiol-mediated dephosphorylation and mitochondrial translocation of Drp1 and cofilin (Fig. 7d). Furthermore, knockdown of ROCK1 abrogated arnidiol-mediated colocalization of Drp1 and cofilin in mitochondria (Fig. 7e). Finally, knockdown of ROCK1 attenuated arnidiol-induced mitochondrial fission and apoptosis (Fig. 7f-i). Taken together, these findings suggest that the activation of ROCK1 is crucial for arnidiol-induced mitochondrial fission and apoptosis and acts by regulating the dephosphorylation and mitochondrial translocation of Drp1 and cofilin.

### Arnidiol inhibits tumor growth in an MDA-MB-231 xenograft mouse model

**Fig. 8:**
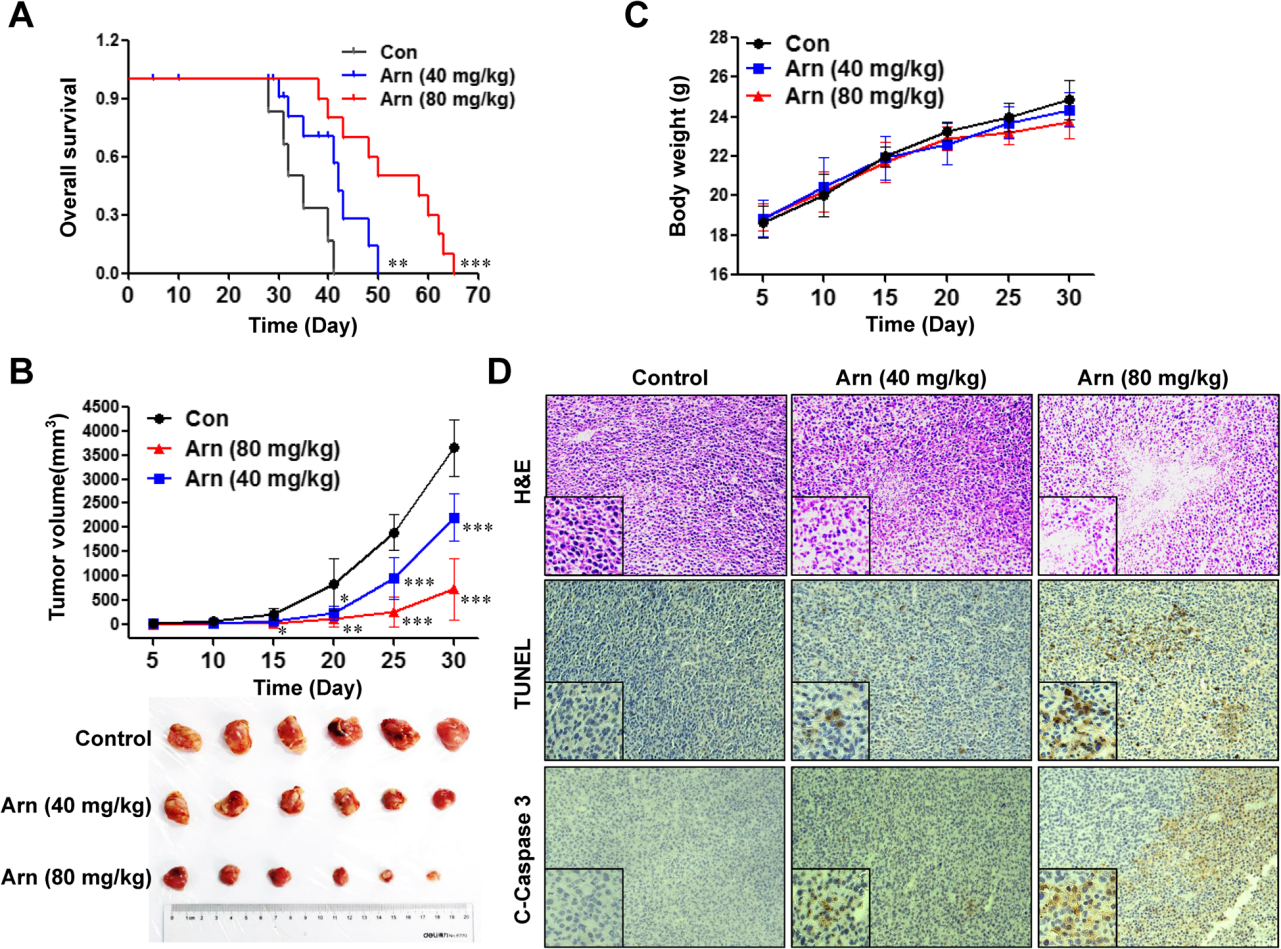
Arnidiol inhibits tumor growth in a MDA-MB-231 xenograft mouse model. **a** Comparison of the overall survival of mice between vehicle, Arn (40 mg/kg) and Arn (80 mg/kg) (*n* = 10 mice per group). Statistical significance in survival was determined by log-rank test. ^**^P < 0.01 or ^***^P < 0.001 compared with control. **b** Average tumor volume in mice treated with vehicle, Arn (40 mg/kg) and Arn (80 mg/kg) (*n* = 6 mice per group). ^*^P < 0.05, ^**^P < 0.01 or ^***^P < 0.001 compared with control. **c** Body weight of mice during the 30 days of treatment. **d** Tumor tissues were sectioned and subjected to H&E, TUNEL, and immunohistochemistry analyses for determination of morphology, apoptosis, and the expression of C-Caspase 3

To determine whether our in vitro findings could be applicable in vivo, nude mice were inoculated subcutaneously with MDA-MB-231 cells followed by injections of vehicle or arnidiol (40 and 80 mg/kg, i.p.) for 70 days starting 1 week after tumor inoculation. Compared with vehicle treatment, daily arnidiol treatment significantly prolonged animal survival (*P* < 0.01) (Fig. 8a). We also examined the effects of arnidiol on the tumor volume of MDA-MB-231 xenografts. Arnidiol modestly suppressed tumor growth at 15 days after drug exposure (^*^P < 0.05 vs vehicle control). This effect became more apparent after 20 and 25 days of drug exposure and was quite extensive after 30 days of drug exposure (^**^P < 0.01 vs vehicle control) (Fig. 8b). However, no statistically significant changes in body weight were noted between the vehicle-treated and arnidiol-treated mice (Fig. 8c).

To evaluate the effects of arnidiol on morphological changes and the induction of apoptosis in tumor sections from MDA-MB-231 xenografts, hematoxylin and eosin (H&E) staining, TUNEL staining, and immunohistochemistry analyses were performed. The sections of MDA-MB-231 xenografts from mice treated with arnidiol had low numbers of cancer cells and exhibited signs of necrosis and infiltration of inflammatory cells (e.g., phagocytic cells) and apoptotic regions (Fig. 8d, top panels). Treatment of mice with arnidiol also resulted in a striking induction of apoptosis in the tumor cells (Fig. 8d, middle panels). Finally, treatment with arnidiol increased the immunoreactivity for cleaved caspase-3, which was indicative of apoptosis (Fig. 8d, bottom panels). These findings suggest that arnidiol inhibits tumor growth in an MDA-MB-231 xenograft mouse model through the induction of apoptosis.

## Discussion

The present results indicate that arnidiol efficiently induced apoptosis in human cancer cells by triggering mitochondrial fission and that this process was due primarily to the interaction and recruitment of Drp1 and cofilin to mitochondria via the activation of ROCK1 signaling. Drp1, a member of the dynamin family of GTPases, is the key component of the mitochondrial fission machinery. During apoptosis, Drp1 is translocated from the cytosol to the fission site of the mitochondria, leading to cytochrome c release and caspase activation [41]. Drp1 activity is regulated by the opposing effects of phosphorylation at two key serines. Phosphorylation of serine 616 increases Drp1 activity, whereas phosphorylation of serine 637 decreases it [7]. Consistent with these reports, our findings demonstrated that during arnidiol-induced apoptosis, dephosphorylated Drp1 (Ser637) can translocate to the mitochondria, leading to mitochondrial fission. First, arnidiol treatment decreased the phosphorylation of Drp1 (Ser637), whereas it did not change the phosphorylation of Drp1 (Ser616). Second, overexpression of Drp1 S637A (a dephosphomimetic) promoted the mitochondrial translocation of Drp1 in arnidiol-treated cells, whereas overexpression of Drp1 S637D (a phosphomimetic) reduced the mitochondrial translocation of Drp1. Third, overexpression of Drp1 S637A promoted mitochondrial fission and apoptosis in arnidiol-treated cells, whereas overexpression of Drp1 S637D reduced arnidiol-mediated mitochondrial fission and apoptosis. Thus, our data indicate that dephosphorylation of Drp1 at Ser 637 is required for arnidiol-induced mitochondrial translocation of Drp1, mitochondrial fission, and apoptosis.

In this study, we also found that dephosphorylation of cofilin (Ser3) is crucial for mitochondrial translocation of cofilin, mitochondrial fission, and apoptosis in arnidiol-treated cells. A recent study indicated that mitochondrial translocation of cofilin is an early step in mitochondrial fission and apoptosis [13, 42]. Only dephosphorylated cofilin can translocate the fission site of mitochondria, leading to mitochondrial fission and apoptosis [13]. Consistent with this report, the dephosphorylation and mitochondrial translocation of cofilin are necessary for arnidiol-induced mitochondrial fission and apoptosis based on the following findings. First, the mitochondrial translocation of cofilin occurs in arnidiol-treated cells. Second, arnidiol treatment reduced the phosphorylation of cofilin (Ser3). Third, overexpression of cofilin S3A (a dephosphomimetic) promoted the mitochondrial translocation of cofilin in arnidiol-treated cells, whereas overexpression of cofilin S3E (a phosphomimetic) reduced the mitochondrial translocation of cofilin. Fourth, overexpression of cofilin S3A promoted mitochondrial fission and apoptosis in arnidiol-treated cells, whereas overexpression of cofilin S3E reduced mitochondrial fission and apoptosis. Thus, our findings indicate that dephosphorylation of cofilin (Ser3) seems to be an essential step for the mitochondrial translocation of cofilin, mitochondrial fission, and apoptosis in response to arnidiol treatment.

Surprisingly, we found that the interaction and colocalization of Drp1 and cofilin is involved in arnidiol-induced mitochondrial fission and apoptosis. A recent study revealed that cofilin1-dependent actin dynamics control Drp1-mediated mitochondrial fission [38]. This study unraveled a novel function for cofilin-dependent actin dynamics in mitochondrial fission and identified cofilin as a negative regulator of mitochondrial Drp1 activity. In contrast to this report, our results suggest the identical roles of Drp1 and cofilin in arnidiol-induced mitochondrial fission and apoptosis. First, both Drp1 and cofilin translocated from the cytosol to the mitochondria during arnidiol-induced mitochondrial fission. Second, interaction and colocalization of Drp1 and cofilin at the outer mitochondrial membrane occur in arnidiol-treated cells. Third, knockdown of either Drp1 or cofilin attenuated the interaction and colocalization between cofilin and Drp1 and attenuated mitochondrial fission and apoptosis. To the best of our knowledge, this is the first report to demonstrate that the recruitment and interaction of Drp1 and cofilin in mitochondria seem to be essential for arnidiol-induced mitochondrial fission and apoptosis.

The present study demonstrates that the activation of ROCK1 plays an essential role in regulating the dephosphorylation and mitochondrial translocation of Drp1 and cofilin. ROCK1 belongs to a family of serine/threonine kinases that are activated via interaction with Rho GTPases. A number of ROCK1 targets have been identified, most of which are phosphatases and are involved in the regulation of cytoskeletal dynamics, cell morphology, and contraction [18, 43, 44]. Recent studies have shown that ROCK1 plays a critical role in the regulation of mitochondrial translocation of Drp1 and cofilin during the induction of apoptosis [23, 45]. One study showed that hyperglycemia-induced mitochondrial fission depends on both ROCK1 activation and Drp1 translocation to the mitochondria. This ROCK1-dependent metabolic pathway involves phosphorylation of Drp1 at Ser600, which promotes its recruitment to the mitochondria [40]. A number of studies have shown that ROCK1 activation can regulate the activation/dephosphorylation of cofilin by inducing PP1 and PP2A phosphatase activities or controlling the phosphorylation of cofilin by LIM kinase [39, 46]. The bulk of evidence suggests that ROCK1 activation plays an important functional role in regulating dephosphorylation and mitochondrial translocation of Drp1 and cofilin during arnidiol-induced mitochondrial fission and apoptosis. First, the activation of ROCK1 and induction of PP1 and PP2A phosphatase activities occur in arnidiol-treated cells. Second, knockdown of ROCK1 by siRNA attenuates arnidiol-mediated Drp1 and cofilin dephosphorylation and mitochondrial translocation. Third, knockdown of ROCK1 attenuates the colocalization of Drp1 and cofilin in mitochondria in arnidiol-treated cells. Fourth, knockdown of ROCK1 attenuates arnidiol-induced mitochondrial fission and apoptosis.

## Conclusions

In summary, the present findings demonstrate for the first time that arnidiol induces mitochondrial fission and apoptosis in human cancer cells. These findings support a hypothetical model of arnidiol-mediated apoptosis in cancer cells in which arnidiol-induced ROCK1 activation represents a primary event resulting in the dephosphorylation of Drp1 (Ser637) and cofilin (Ser3), leading, in turn, to the mitochondrial translocation of Drp1 and cofilin and culminating in mitochondrial fission and apoptosis.

## Data Availability

All data generated or analyzed during this study are included in this published article and its supplementary information files.

## Abbreviations

Arn: Arnidiol
Drp1: Dynamin-related protein 1
Fis1: Mitochondrial fission protein 1
MFF: Mitochondrial fission factor
Mfn1: Mitofusin 1
Mfn2: Mitofusin 2
OPA1: Optic atrophy 1
PP1: Phosphatases type 1
PP2A: Phosphatases type 2A
ROCK1: Rho-associated coiled-coil containing protein kinase1
